# Supplementary material for: LRET-derived HADDOCK structural models describe the conformational heterogeneity required for DNA cleavage by the Mre11-Rad50 DNA damage repair complex
Source: eLife. 2022 Jan 27;11:e69579. doi: 10.7554/eLife.69579 (PMC8824468; doi:10.7554/eLife.69579)
Supplement: Supplementary file 1. — Each row represents data from a unique LRET pair. Distances (in Å) were calculated from the decay of donor-sensitized Bodipy or Cy3 fluorescence emission as described in Materials and methods. Errors are the standard deviation of n ≥ 3 LRET measurements. [file elife-69579-supp1.docx]

Supplementary Table 1: MR^NBD^ LRET probe pair distances

| **Bodipy position** | **Cy3 position** | **Tb^3+^ position** | **3QKU Cβ-Cβ distance** | **LRET**  **Closed distance** | **LRET Partially open distance** | **LRET**  **Open distance** |
| --- | --- | --- | --- | --- | --- | --- |
|  |  |  |  |  |  |  |
| S13C |  | S13C | 42.2 | 35.4 ± 0.3 | 51.1 ± 1.0 |  |
|  | S13C | S13C | 42.2 |  | 50.9 ± 1.1 | 77.8 ± 1.7 |
| S13C |  | L51C | 41.8 | 36.7 ± 1.1 | 51.4 ± 0.9 |  |
| L51C |  | S13C | 41.8 | 34.4 ± 1.1 | 51.2 ± 0.5 |  |
|  | S13C | L51C | 41.8 |  | 51.6 ± 0.3 | 79.7 ± 3.0 |
|  | L51C | S13C | 41.8 |  | 51.3 ± 0.3 | 78.9 ± 1.9 |
|  | A66C | S13C | 52.2 | 52.7 ± 1.2 | 75.4 ± 1.0 |  |
|  | S13C | S93C | 54.6 | 50.3 ± 0.7 | 76.8 ± 0.5 |  |
|  | S93C | S13C | 54.6 | 49.7 ± 1.7 | 79.5 ± 1.5 |  |
| S13C |  | N774C | 33.9 | 37.0 ± 0.9 | 48.7 ± 0.5 |  |
|  | S13C | N774C | 33.9 |  | 44.9 ± 1.4 | 81.0 ± 0.5 |
| V866C |  | S13C | 38.5 | 37.0 ± 0.1 | 49.2 ± 0.3 |  |
| S13C |  | V866C | 38.5 | 34.0 ± 0.7 | 50.2 ± 0.5 |  |
|  | S13C | V866C | 38.5 |  | 48.7 ± 1.8 | 80.4 ± 3.3 |
| L51C |  | L51C | 37.6 | 37.7 ± 0.6 | 45.4 ± 1.1 |  |
|  | L51C | L51C | 37.6 |  | 47.0 ± 1.0 | 78.0 ± 2.0 |
| A66C |  | L51C | 48.6 | 49.3 ± 1.3 |  |  |
| L51C |  | A66C | 48.6 | 52.9 ± 1.7 |  |  |
|  | A66C | L51C | 48.6 |  | 56.3 ± 2.0 | 83.9 ± 4.1 |
|  | L51C | A66C | 48.6 |  | 55.9 ± 1.2 | 80.6 ± 3.9 |
| S93C |  | L51C | 47.5 | 48.1 ± 0.8 |  |  |
| L51C |  | S93C | 47.5 | 51.6 ± 0.4 |  |  |
|  | S93C | L51C | 47.5 |  | 55.1 ± 2.1 | 80.4 ± 3.4 |
|  | L51C | S93C | 47.5 |  | 53.1 ± 2.0 | 83.2 ± 1.1 |
| N774C |  | L51C | 29.8 | 30.9 ± 0.2 | 51.5 ± 0.4 |  |
| L51C |  | N774C | 29.8 | 32.2 ± 1.0 | 50.6 ± 1.6 |  |
|  | N774C | L51C | 29.8 |  | 49.6 ± 1.5 | 80.8 ± 1.8 |
|  | L51C | N774C | 29.8 |  | 47.1 ± 1.4 | 83.0 ± 0.6 |
| V866C |  | L51C | 46.8 | 47.6 ± 0.7 |  |  |
| L51C |  | V866C | 46.8 | 49.0 ± 0.3 |  |  |
|  | V866C | L51C | 46.8 |  | 52.4 ± 1.9 | 81.3 ± 2.5 |
|  | L51C | V866C | 46.8 |  | 52.2 ± 3.4 | 79.7 ± 1.7 |
| A66C |  | A66C | 61.3 | 53.7 ± 1.9 |  |  |
|  | A66C | A66C | 61.3 | 49.2 ± 1.3 | 81.5 ± 1.9 |  |
|  | A66C | S93C | 61.6 | 48.5 ± 2.1 | 78.7 ± 1.2 |  |
|  | S93C | A66C | 61.6 | 47.1 ± 1.8 | 82.1 ± 2.5 |  |
| A66C |  | V866C | 49.2 | 50.4 ± 1.3 |  |  |
|  | V866C | A66C | 49.2 |  | 55.0 ± 1.5 | 83.1 ± 2.0 |
|  | A66C | V866C | 49.2 |  | 53.7 ± 1.7 | 79.5 ± 2.4 |
| A66C |  | N774C | 34.8 | 34.7 ± 0.6 | 49.3 ± 1.2 |  |
|  | A66C | N774C | 34.8 |  | 47.2 ± 0.5 | 82.8 ± 1.3 |
| S93C |  | S93C | 58.8 | 52.0 ± 1.2 |  |  |
|  | S93C | S93C | 58.8 | 51.4 ± 1.9 | 79.9 ± 0.6 |  |
| S93C |  | N774C | 31.3 | 32.6 ± 0.6 | 49.9 ± 1.0 |  |
| N774C |  | S93C | 31.3 | 34.2 ± 1.1 | 50.6 ± 0.6 |  |
|  | N774C | S93C | 31.3 |  | 45.6 ± 1.0 | 75.6 ± 1.2 |
|  | V866C | S93C | 56.5 | 52.4 ± 1.5 | 80.1 ± 1.0 |  |
|  | S93C | V866C | 56.5 | 48.3 ± 2.7 | 78.8 ± 2.7 |  |
| N774C |  | N774C | 36.7 | 35.7 ± 0.2 | 51.5 ± 2.3 |  |
|  | N774C | N774C | 36.7 |  | 45.8 ± 1.8 | 83.5 ± 2.2 |
| V866C |  | V866C | 37.0 | 36.2 ± 1.5 | 53.5 ± 0.8 |  |
|  | V866C | V866C | 37.0 |  | 50.6 ± 1.8 | 82.7 ± 3.3 |

Each row represents data from a unique LRET pair. Distances (in Å) were calculated from the decay of donor-sensitized Bodipy or Cy3 fluorescence emission as described in Methods. Errors are the standard deviation of n ≥ 3 LRET measurements.
