# Supplementary material for: LRET-derived HADDOCK structural models describe the conformational heterogeneity required for DNA cleavage by the Mre11-Rad50 DNA damage repair complex
Source: eLife. 2022 Jan 27;11:e69579. doi: 10.7554/eLife.69579 (PMC8824468; doi:10.7554/eLife.69579)
Supplement: Supplementary file 2. — The average experimental LRET distance measured for each pair of Rad50 LRET probes was used as unambiguous restraints in closed, partially open, and open HADDOCK simulations. The three resulting HADDOCK models had the reported Cβ-Cβ distance between indicated probe positions. [file elife-69579-supp2.docx]

Supplementary Table 2: MR^NBD^ LRET experimental probe distances and HADDOCK model distances

| **Pair of Rad50 residues** | **3QKU**  **dimer**  **Cβ-Cβ distance (Å)** | **LRET unambiguous restraint (Å)** | | | **HADDOCK model Cβ-Cβ distance (Å)** | | |
| --- | --- | --- | --- | --- | --- | --- | --- |
|  |  | **Closed**  **( ±5)** | **Partially open**  **( ±5)** | **Open**  **( ±7)** | **Closed** | **Partially open** | **Open** |
| S13-S13 | 42.2 | 35.4 | 51.0 | 77.8 | 40.4 | 56.2 | 74.9 |
| L51-L51 | 37.6 | 37.7 | 46.2 | 78.0 | 36.1 | 51.6 | 82.0 |
| A66-A66 | 61.3 | 51.5 | 81.5 |  | 58.1 | 74.4 | 93.0 |
| S93-S93 | 58.8 | 51.7 | 79.9 |  | 56.2 | 73.4 | 97.0 |
| N774-N774 | 36.7 | 35.7 | 48.7 | 83.5 | 38.4 | 50.0 | 78.3 |
| V866-V866 | 37.0 | 36.2 | 52.1 | 82.7 | 36.1 | 46.9 | 73.4 |
| S13-L51 | 41.8 | 35.6 | 51.4 | 79.3 | 40.0 | 53.6 | 79.1 |
| S13-A66 | 52.2 | 52.7 | 75.4 |  | 49.5 | 65.7 | 84.0 |
| S13-S93 | 54.6 | 50.0 | 78.2 |  | 52.3 | 67.4 | 88.1 |
| S13-N774 | 33.9 | 37.0 | 46.8 | 81.0 | 32.9 | 41.8 | 73.2 |
| S13-V866 | 38.5 | 35.5 | 49.4 | 80.4 | 37.1 | 52.8 | 74.0 |
| L51-A66 | 48.6 | 51.1 | 56.1 | 82.3 | 46.4 | 61.2 | 86.2 |
| L51-S93 | 47.5 | 49.9 | 54.1 | 81.8 | 45.4 | 62.3 | 88.8 |
| L51-N774 | 29.8 | 31.6 | 49.7 | 81.9 | 29.2 | 44.4 | 77.1 |
| L51-V866 | 46.8 | 48.3 | 52.3 | 80.5 | 46.0 | 57.1 | 82.7 |
| A66-S93 | 61.6 | 47.8 | 80.4 |  | 58.8 | 74.7 | 95.8 |
| A66-N774 | 34.8 | 34.7 | 48.3 | 82.8 | 32.9 | 46.7 | 77.5 |
| A66-V866 | 49.2 | 50.4 | 54.3 | 81.3 | 47.3 | 63.4 | 83.1 |
| S93-N774 | 31.3 | 33.4 | 48.7 | 75.6 | 29.4 | 49.2 | 79.8 |
| S93-V866 | 56.5 | 50.4 | 79.5 |  | 54.9 | 69.7 | 90.0 |

The average experimental LRET distance measured for each pair of Rad50 LRET probes was used as unambiguous restraints in closed, partially open, and open HADDOCK simulations. The three resulting HADDOCK models had the reported Cβ-Cβ distance between indicated probe positions.
